# Genetic elements associated with antimicrobial resistance among avian pathogenic *Escherichia coli*

**DOI:** 10.1186/s12941-016-0174-9

**Published:** 2016-11-25

**Authors:** Amal Awad, Nagah Arafat, Mohamed Elhadidy

**Affiliations:** 1 Department of Bacteriology, Mycology and Immunology, Faculty of Veterinary Medicine, Mansoura University, Mansoura, 35516 Egypt; 2 Department of Poultry diseases, Faculty of Veterinary Medicine, Mansoura University, Mansoura, 35516 Egypt; 3 Foodborne Pathogens, Scientific Institute of Public Health, Juliette Wytsmanstraat 14, 1050 Brussels, Belgium

**Keywords:** Antimicrobial resistance, *E. coli*, Poultry, Integron, Genes

## Abstract

**Background:**

Avian-pathogenic *Escherichia coli* (APEC) are pathogenic strains of *E. coli* that are responsible for one of the most predominant bacterial disease affecting poultry worldwide called avian colibacillosis. This study describes the genetic determinants implicated in antimicrobial resistance among APEC isolated from different broiler farms in Egypt.

**Methods:**

A total of 116 APEC were investigated by serotyping, antimicrobial resistance patterns to 10 antimicrobials, and the genetic mechanisms underlying the antimicrobial-resistant phenotypes.

**Results:**

Antibiogram results showed that the highest resistance was observed for ampicillin, tetracycline, nalidixic acid, and chloramphenicol. The detected carriage rate of integron was 29.3% (34/116). Further characterization of gene cassettes revealed the presence gene cassettes encoding resistance to trimethoprim (*dfrA1*, *dfrA5*, *dfrA7*, *dfrA12*), streptomycin/spectinomycin (*aadA1*, *aadA2*, *aadA5, aadA23*), and streptothricin (*sat2*). To our knowledge, this the first description of the presence of *aadA23* in APEC isolates. Analysis of other antimicrobial resistance types not associated with integrons revealed the predominance of resistance genes encoding resistance to tetracycline (*tetA* and *tetB*), ampicillin (*bla*_TEM_), chloramphenicol (*cat1*), kanamycin (*aphA1*), and sulphonamide (*sul1* and *sul2*). Among ciprofloxacin-resistant isolates, the S83L mutation was the most frequently substitution observed in the quinolone resistance-determining region of *gyrA* (56.3%). The *bla*_TEM_ and *bla*_CTX−M−1_ genes were the most prevalent among APEC isolates producing extended-spectrum beta-lactamase (ESβL).

**Conclusions:**

These findings provided important clues about the role of integron-mediated resistance genes together with other independent resistance genes and chromosomal mutations in shaping the epidemiology of antimicrobial resistance in *E. coli* isolates from poultry farms in Egypt.

## Background

Avian colibacillosis is an extraintestinal infection associated with upper respiratory tract infection typically air saculitis that can evolve into multiple lesions in different organs as polyserositis, cellulitis, salpingitis, perihepatitis, peritonitis, septicaemia, and death. These cause severe economic losses in the poultry industry due to the remarkable number of morbidities, mortalities, slaughter condemnation, and reduced productivity of the affected birds [1].

To reduce the high incidence and mortalities caused by avian colibacillosis in poultry farms, antimicrobials are considered as one of the gold choices among veterinarians in the poultry industry. However, in Egypt and other developing countries, antimicrobials are not only used for therapeutic reasons but also for prophylactic purposes in disease prevention and growth promotion. Such overuse and/or misuse contribute to the development and spread of antimicrobial resistance (AMR) among poultry flocks, leading to emergence of multiple drug resistance (MDR) pathogens [2]. These MDR profiles can be transmitted to human through the food chain adding serious burden to human health. In addition, several studies supported the growing evidence that antimicrobial resistance can occur in the absence of selective pressure, highlighting the crucial role of antibiotic resistance genes in the development of MDR bacteria [3]. The acquisition and dissemination of these antibiotic resistance genes is mainly mediated by horizontal gene transfer in which several mobile genetic elements facilitate this process, including plasmids, transposons, integrons, and bacteriophages [4]. Integron is one of the major genetic elements by which bacteria can capture or excise one or more resistance gene cassettes by site-specific recombination. The mobilization of integron through transposons, plasmids, or other mobile genetic elements enables the spread of integrated resistance genes. This potentially facilitate the integration of almost all antimicrobial agents through the integron and increase the diversity of integrons in isolates from different sources, including human, avian, livestock, and environmental isolates [4–6].

The aim of this study was to determine the antibiotic susceptibility phenotypes of APEC isolated from different broiler farms in Egypt and to evaluate the association of observed phenotypes with the acquisition of integron mediated resistance gene cassettes or other resistance genes and chromosomal mutations located outside the integron.

## Methods

### Bacterial isolates

In total, 116 APEC isolates recovered from 400 different samples were assayed. Samples were obtained from liver, lungs, air sacs and spleen from chicken with typical lesions of colisepticemia and were collected from 28 different broiler farms located in different geographic areas of Dakahlia Governorate in 2014 and 2015. Samples were cultured onto eosin methylene blue (EMB) agar (Oxoid, Basingstoke, UK) and incubated for 18–24 h at 37 °C. Typical colonies were subcultured onto MacConkey agar (Oxoid, Basingstoke, UK) and were biochemically confirmed using API 20E system (BioMérieux, Marcy-l’Étoile, France). Once identified, the isolates were preserved at −70 °C in brain heart infusion broth containing 20% glycerol (vol/vol) for further studies. Serotyping of *E. coli* isolates was performed using rapid diagnostic *E. coli* antisera sets (Denka Seiken Co., Japan) using 8 polyvalent, 43 monovalent somatic, and 22 flagellar antisera. Bacterial DNA for PCR analysis was prepared by boiling a bacterial culture in 300 µl of distilled water for 10 min, followed by centrifugation for 5 min at 10,000×*g*. A volume of 1 µl of the supernatant was used as a template for each 25 µl PCR mixture.

### Antimicrobial susceptibility testing and detection of ESβL

The susceptibility of isolates to 10 different antimicrobial agents was determined by disc diffusion assay as described by Clinical and Laboratory Standards Institute (CLSI) [7]. According to the measurement of inhibition zones, tested strains were evaluated as susceptible or resistant. The following antimicrobial discs (Oxoid, Basingstoke, UK) were used: gentamicin (GN, 10 μg), kanamycin (KAN, 30 μg), ampicillin (AMP, 10 μg), cefotaxime (CTX, 30 μg), nalidixic acid (NA, 30 μg), ciprofloxacin (CIP, 5 μg), tetracycline (TET, 30 μg), sulfamethoxazole-trimethoprim (STX, 25 μg), chloramphenicol (C, 30 μg), and streptomycin (S, 10 μg). The isolates were defined as multidrug-resistant APEC if they exhibited resistance to at least one agent in three or more antimicrobial categories [8]. *Escherichia coli* strain ATCC 25922 was used as a reference strain for susceptibility test. All cefotaxime-resistant isolates were confirmed for the production of extended-spectrum beta-lactamase (ESβL) by using the double-disk synergy test (DDST) following recommendation and interpretations of CLSI standards [7].

### Detection of integrons and characterization of associated gene cassettes

The presence of integrons among APEC isolates was assayed using primers hep35 and hep36 that amplify conserved regions of integron-encoded integrase genes *intI1*, *intI2*, and *intI3* using published PCR protocols [9]. Integron-positive isolates were characterized for the integron class using integron class-specific primers and PCR conditions previously described [10]. Further characterization of gene cassette content within integron classes was determined using primers 5′CS/3′CS and hep74/hep51 for amplification of gene cassettes within integron classes 1 and 2, respectively as published [11, 12]. Samples with similar amplicon size were further subjected to restriction enzyme *HinfI* or *RsaI* [12] following manufacturer instructions (New England Biolabs, UK) and fragments obtained were separated on a 2.5% agarose gel. Isolates with similar polymorphism patterns were considered to harbor similar gene cassettes. Amplification products of samples with unique amplification products and one representative sample from samples with similar restriction patterns were purified from the agarose gel using QIA quick Gel Extraction Kit (Qiagen, USA) and sequenced using ABI Prism 377 automated sequencer (Applied Biosystems). All sequence results were compared with the available sequences in GenBank (http://www.ncbi.nlm.nih.gov/BLAST).

### Detection of antimicrobial resistance genes outside the integron cassettes

Different PCR protocols were applied to detect specific genetic determinants corresponding to the resistance phenotype using Applied Biosystems 96-well thermal cycler and PCR conditions suggested by the referenced authors. These determinants included those encoding resistance to tetracycline (*tetA*, *tetB*, *tetC*, *tetD*, *tetE*, and *tetG*) [13], chloramphenicol (*cat1*, *cat2*, *cat3*, *cmlA*, and *cmlB*) [14], streptomycin (*strA*-*strB*) [15], Kanamycin (*aphA1* and *aphA2*) [16], and sulfonamide including *sul1*, *sul2* [17], and *sul3* [18]. The PCR amplification products were separated by agarose gel electrophoresis and visualized by ethidium bromide staining. Resistance to ciprofloxacin was assayed by determining mutations in DNA gyrase subunit A (*gyrA*) and topoisomerase IV subunit C (*ParC*) in the quinolone resistance determining region (QRDR) using protocol previously described [19]. The resulting PCR amplicons were purified using QIA quick Gel Extraction Kit (Qiagen, USA) and bi-directionally sequenced using same primers. Strains that exhibited negative results for mutations in QRDR were screened for plasmid-mediated quinolone resistance (PMQR) including *qnrA*, *qnrB*, *qnrS* [20], *qnrD* [21], and *aac (6′)*-*Ib*-*cr* [22]. Ampicillin-resistant isolates were screened for the β-Lactamases genes *bla*_TEM_ and *bla*_SHV_, and *bla*_OXA−1_ using multiplex PCR protocol published [23]. The later protocol was used to screen potential ESβL-producing *E. coli* isolates that initially exhibited resistance to cefotaxime. These isolates were further screened for *bla*_CTX-M_ five phylogenetic groups (groups 1, 2, 8, 9, and 25) using multiplex PCR protocol previously described [24].

### Pulsed-field gel electrophoresis (PFGE)

PFGE was performed to determine that isolates exhibiting similar antibiogram resistance profile and similar serotype are non-identical. PFGE for tested APEC isolates was performed using XbaI restriction enzyme according to the PulseNet protocol from the Centers for Disease Control and Prevention [25]. At least one band difference was required to distinguish between pulsotypes.

### Statistical analysis

Differences in the occurrence of integrons in resistant and susceptible APEC strains were tested using the Chi square (*χ* ^2^) test. Differences among means with *P* < 0.05 were considered as statistically significant. For the statistical analysis, intermediate resistant strains were considered as resistant.

## Results

### APEC serotypes

A total of 116 *E. coli* isolates were recovered from 400 samples from diseased chicken. Serotyping recovered isolates identified 15 different *E. coli* serotypes (Table 1). All isolates exhibiting similar serotype and antimicrobial resistance patterns were confirmed to be non-duplicates as confirmed by PFGE. Among identified serotypes, O78 was found to be the most prevalent serotype (27.6%).

**Table 1:** Overview and characteristics (MDR strains, integron presence, and antibiotic resistance) of the APEC strains classified per serotype

Serotype	No. of strains	No. of MDR strains^a^	No. of different antibiotic resistance profiles	No. isolates carrying integrons		
				Class 1	Class 2	Total
O1:H7	14 (12.1%)	6	14	2	0	2 (14.3%)
O2:H6	18 (15.5%)	14	18	6	0	6 (33.3%)
O8	2 (1.7%)	2	2	0	0	0 (0%)
O111:H2	4 (3.4%)	4	4	2	2	4 (100%)
O26:H11	6 (5.2%)	6	6	4	0	4 (66.7%)
O44:H18	2 (1.7%)	2	2	0	0	0 (0%)
O55:H7	10 (8.6%)	8	6	0	0	0 (0%)
O78	32 (27.6%)	28	24	16	0	16 (50.0%)
O119:H6	6 (5.2%)	4	6	0	0	0 (0%)
O124	4 (3.4%)	4	4	0	0	0 (0%)
O126:H2	6 (5.2%)	6	6	0	0	0 (0%)
O127:H6	6 (5.2%)	4	6	0	2	2 (33.3%)
O128:H2	2 (1.7%)	2	2	0	0	0 (0%)
O142:H6	2 (1.7%)	0	2	0	0	0 (0%)
O158	2 (1.7%)	0	2	0	0	0 (0%)
Total	116	90 (77.6%)		30 (25.9%)	4 (3.4%)	34 (29.3%)

^a^ *MDR strains* multidrug-resistant strains that exhibited resistance to 3 or more different classes of antimicrobials

### Antimicrobial resistance patterns and ESβL phenotype

Antibiotic susceptibility of 116 APEC isolates against 10 different antimicrobial drugs is presented in Table 2. The highest frequencies of resistance detected were those against ampicillin (100%), tetracycline (93.1%), nalidixic acid (84.5%), chloramphenicol (84.5%), kanamycin (69.0%), sulfamethoxazole-trimethoprim (58.6%), cefotaxime (58.6%), streptomycin (50.0%), gentamicin (48.3%), and ciprofloxacin (41.4%). A diversity of resistance patterns observed among tested *E. coli* isolates is shown in Table 1. Screening the potential ESβL producers confirmed that all cefotaxime-resistant isolates to be ESβL producers (68/68, 100%).

**Table 2:** Molecular characterization of antimicrobial resistance genotypes among APEC isolates

Antibiotic	Antimicrobial class	Number of resistance isolates	Associated genes tested	Number of positive isolates
Ampicillin	Penicillins	116 (100%)	*bla*_TEM_	102
			*bla*_SHV_	0
			*bla*_oxa1_	14
Chloramphenicol	Phenicols	98 (84.5%)	*cat1*	86
			*cat2*	4
			*cat3*	0
			*cmlA*	8
			*cmlB*	0
Streptomycin	Aminoglycosides	58 (50%)	*strA*-*strB*	34
Ciprofloxacin	Quinolones	48 (41.4%)	PMQR^a^	
			*qnrA*	10
			*qnrB*	4
			*qnrS*	2
			*qnrD*	0
			*aac (6′)*-*Ib*-*cr*	0
			QRDR^b^	
			S83L (*gyrA*)	27
			S83L + D87 N (*gyrA*)	4
			S80I (*pyrC*)	1
Sulfamethoxazole-trimethoprim	Potentiated sulfonamides	68 (58.6%)	*sul1*	23
			*sul2*	39
			*sul3*	0
Tetracycline	Tetracyclines	108 (93.1%)	*tetA*	50
			*tetB*	54
			*tetC*	0
			*tetD*	0
			*tetE*	0
			*tetG*	0
			*tetA* + t*etB*	4
Kanamycin	Aminoglycosides	80 (69.0%)	*aphA1*	65
			*aphA2*	11
			*aphA1* + *aphA2*	4
Cefotaxime (ESβL)	Cephalosporins	68 (58.6%)	CTX-M (group 1)	15
			CTX-M (group 2)	0
			CTX-M (group 8)	0
			CTX-M (group 9)	2
			CTX-M (group 25)	0
			*bla*_TEM_	20
			*bla*_SHV_	0
			*bla*_oxa1_	7
			CTX-M (group 1) + *bla*_TEM_	24

^a^Plasmid-mediated quinolone resistance

^b^Quinolone resistance determining region

### Distribution of integrons and associated gene cassettes

Of the 116 isolates tested, 34 isolates (29.3%) representing six serotypes were integron positive (Table 1). All integron-harboring *E. coli* isolates were multidrug resistant. Integron-positive isolates were significantly more resistant to streptomycin, kanamycin, gentamicin, and sulfamethoxazole-trimethoprim than integron-negative isolates (P < 0.05). Among integron-positive isolates, class 1 integrons were detected in 30 isolates (25.9%) and class 2 in four isolates (3.4%) (Table 1). None of the isolates possessed the combination of class 1 and class 2 integrons. Class 3 was absent in tested APEC isolates. Further characterization of gene cassettes by PCR, restriction digestion, and sequencing revealed eight different cassette arrangements within class 1 integron and a single cassette arrangement within class 2 integron. Among class 1 integron, the gene cassettes included those encoding resistance to trimethoprim (*dfrA1*, *dfrA5*, *dfrA7*, and *dfrA12*) and streptomycin (*aadA1*, *aadA2*, *aadA5,* and *aadA23*). A total of seven isolates harbored *aadA1* + *dfrA1* (20.6%); six isolates carried *dfrA7* (17.6%); five isolates had *aadA1* (14.7%); four isolates carried *aadA5* (11.8%); three isolates (8.8%) contained *dfrA5*; two isolate (5.8%) carried *aadA2* + *dfrA12*, two isolate (5.8%) carried *aadA2*3, and one (2.9%) had *dfrA1*. All class 2 integron-carrying isolates possessed the gene cassette array *dfrA1*-*sat2*-*aadA1* that encode resistance to trimethoprim, streptothricin, and streptomycin (Table 3).

**Table 3:** Distribution of class 1 and class 2 integrons among APEC isolates

Integron genotype	Integron class	Number of positive integron harboring isolates
*aadA1*-*dfrA1*	1	7
*dfrA7*	1	6
*aadA1*	1	5
*aadA5*	1	4
*dfrA5*	1	3
*aadA2*-*dfrA12*	1	2
*aadA2*3	1	2
*dfrA1*	1	1
*dfrA1*-*sat2*-*aadA1*	2	4
Total		34

### Distribution of antimicrobial resistance genes outside the integron cassettes

Different frequencies of genes encoding resistance to tetracycline, chloramphenicol, ampicillin, streptomycin, kanamycin, ciprofloxacin, cefotaxime, sulfonamide, and ESβL production are reported in Table 2. Among tetracycline resistant isolates, 50 (46.3%) isolates carried *tetA* and 54 (50.0%) isolates were positive for *tetB*. Our results also showed that four isolates (3.7%) possessed both determinants. All isolates were negative for *tetC*, *tetD*, *tetE*, and *tetG*. Among the ampicillin-resistant isolates, 102 (87.9%) isolates possessed *bla*_TEM_, 14 (12.1%) isolates carried *bla*_oxa1_. None of these isolates carried *bla*_SHV_ gene. The *cat1* was the most common gene present among the chloramphenicol-resistant isolates (86/98; 87.8%). Isolates (n = 68) that were resistant for sulfamethoxazole were tested for amplification of the *sul* genes. The *sul2 and sul1* genes were present in 39 (57.3%) and 23 (33.8%) isolates, respectively. All ciprofloxacin-resistant APEC isolates were screened for mutations in QRDR of *gyrA* and *ParC* by PCR and DNA sequencing. Our sequencing results showed that a total of 56.3% (27/48) of tested isolates possessed S83L mutation in *gyrA*. Four ciprofloxacin- resistant isolates harbored double mutations of *gyrA* at residues 83 and 87 (S83L + D87 N). One APEC isolate had a single mutation in *parC* encoding Ser80Ile. The PMQR were investigated in APEC isolates with no mutations in QRDR to explore further mechanisms of resistance. The *qnrA*, *qnrB*, and *qnrS* genes were found in these isolates (10, 4 and 2 isolates, respectively). None of the isolates were positive for *qnrD* or *aac (6′)*-*Ib*-*cr* genes. The *strA*-*strB* genes encoding streptomycin resistance were examined to investigate streptomycin resistance determinants other than *aadA* present in the integron cassette and both genes were detected in 34 (58.6%) isolates. The *aphA1* and *aphA2* gene markers, conferring kanamycin resistance were identified in a total of 65 (81.3%) and 11 (13.7%) isolates, respectively. The combined presence of both gene alleles was found in 4 (5%) isolates. Screening ESβL-producing *E. coli* isolates by multiplex PCR analysis revealed that 15 isolates were group in CTX-M-1 group. Group 9 enzymes were produced by two isolates. In addition, *bla*_TEM_ and *bla*_OXA−1_ genes were present in 20 (29.4%) and 7 (10.3%) ESβL-producing *E. coli* isolates, respectively. A total of 24 isolates were observed to simultaneously carry *bla*_TEM_ and contain alleles encoding group 1 CTX-M enzymes (Table 2).

## Discussion

The emerging antimicrobial resistance among different APEC isolates has stimulated our interest in exploring the mechanisms of resistance and the frequencies of genetic determinants. In this study, the majority of recovered isolates belonged to serogroups O1, O2, O78. A similar observation was reported by other studies supporting the previous postulation that these serotypes are the most common serotypes in *E. coli* of avian origin worldwide [26].

Similar to the findings of previous studies [2, 5], most (84–100%) APEC isolates were highly resistant to ampicillin, tetracycline, nalidixic acid, and chloramphenicol. Many (41–69%) APEC isolates were resistant to streptomycin, gentamycin, kanamycin, sulfamethoxazole combined with trimethoprim, ciprofloxacin, and cefotaxime. Most of these antimicrobial agents are regularly used as prophylactic agents or as growth promoters in the poultry industry in Egypt.

In the present study, the proportion of integron carriage among APEC isolates is consistent with previous studies worldwide [10, 27]. In a study conducted in Egypt on APEC isolated from septicemic broilers, higher prevalence was reported for class 1 and 2 (46.6 and 9.6%, respectively) [5]. Our results revealed that the genes found in class 1 and class 2 integrons encode for adenyltransferase conferring resistance to streptomycin and spectinomycin (*aadA*) and for a dihydrofolate reductase conferring resistance to trimethoprim (*dfr*). These gene cassettes are the most frequently detectable genes from pathogenic and commensal *E. coli* from poultry farms and also from different animal, environmental, and human sources [4–6].

Among class 1 integron-harboring isolates, four different trimethoprim resistance genes (*dfrA1*, *dfrA5*, *dfrA7*, *dfrA12*) and four streptomycin/spectinomycin resistance genes (*aadA1*, *aadA2*, *aadA5, aadA23*) were detected as gene cassettes in class 1 integrons. The *aadA23* gene cassette detected in this study was first reported in 2005 in Brazil from *Salmonella* Agona strains isolated from a pig carcass [28]. In Egypt, this gene cassette was previously described among *E. coli* strains isolated from neonatal calf diarrhea [29]. To our knowledge, the first description of *aadA23* in *E. coli* of avian origin. All class 2 integron carrying isolates processed the gene cassette array *dfrA1*-*sat2*-*aadA1* that is considered as the classical gene cassette found in class 2 integron among APEC strains [5, 30]. The absence of class 3 integron among our isolates corroborate previous studies [5, 30], suggesting the less significant role of this integron in dissemination of antimicrobial resistance among APEC.

We further assessed the presence of other genetic determinants located outside the integron that were previously proposed to be responsible for resistance to different antimicrobial agents. Among tetracycline-resistant isolates, *tetA* and *tetB* markers were the only *tet* genes reported in this study either in single or combined genes. These results supported the observation that these efflux genes are the most frequent *tet* genes found in *Enterobacteriaceae* [31]. The high prevalence of these variants might be attributed to their higher association with plasmids, mainly the conjugative ones or transposons [31], suggesting that these genes are the major genes implicated in the efflux mechanism in APEC leading to the resistance phenotype. The high prevalence of *cat1* among chloramphenicol-resistant isolates supports previous studies highlighting the crucial role of this allele in chloramphenicol resistance [16]. The combined presence of *strA*-*strB* determinants encoding two enzymes required for high level of streptomycin resistance [32] was assayed in this study as *aadA* gene observed in the integron has been proposed to confer low level of streptomycin [33]. In this study, the presence of the *strA*-*strB* gene pair among streptomycin-resistant was within the range reported among APEC strains isolated in Korea during the period 2000 to 2005 [34]. The high prevalence of *aphA1* among kanamycin-resistant isolates is consistent with other studies that proposed *aphA1* to be the main neomycin/kanamycin resistance marker [16, 35]. The predominance of *bla*_TEM_ gene (87.9%) among ampicillin-resistant isolates corroborates a previous study that suggested *bla*_TEM_ gene to be the most common β-lactamase responsible for ampicillin resistance [34].

Resistance to quinolone in APEC is mainly mediated by spontaneous mutations in the QRDR of *gyrA* and *parC* genes [2, 34]. Nonetheless, in a previous study conducted among APEC isolates in Egypt, these substitutions in QRDR genes were not screened and only PMQR genes were investigated [5]. Our results revealed that among ciprofloxacin-resistant isolates, the mutation in *gyrA* (S83L) involved in fluoroquinolone resistance was the most prevalent genetic determinant, an observation previously reported in a study conducted among quinolone-resistant APEC isolates in Korea [34].

One of the most striking findings from this study was the high prevalence of ESβL-producing isolates recovered from broilers (58.6%). This represents a serious public health threats attributed to the ability of these bacteria to hydrolyze third-generation cephalosporins that are commonly used to treat serious infections and the potential transfer and spread of these resistance genes to human through food chain, direct contact, or the environment [36–38]. The higher frequency of CTX-M-1-group in this study supports recent reports that postulated this group to the most predominant CTX-M group among ESβL-producing APEC in Egypt or worldwide [5, 39, 40].

## Conclusions

Our results reported high rates of antibiotic resistance among APEC isolates recovered from different poultry farms in Egypt, including those drugs of broad-spectrum-activity such as β-lactams, third generation cephalosporins, and quinolones. Furthermore, our findings suggested diverse genetic makeup involved in antimicrobial resistance phenotype. Indeed, integron-mediated resistance could explain only a part of the resistance profile of the isolates as other independent genes were significantly observed. This diversity in distribution of resistance determinants could be responsible for the high resistance profiles recorded and the potential spread of antimicrobial resistance determinants among APEC. These advices more restriction in antimicrobials use in poultry farms among veterinarians to prevent and control the spread of antimicrobial-resistant bacteria and their genetic determinants. Consequently, our future studies will be directed to study the clonal link and compare the overlapping characteristics of recovered APEC isolates with human ExPEC (extra-intestinal pathogenic *E. coli*) to confirm the zoonotic potential of APEC, including their potential ability to transfer antimicrobial resistance to human.

## Abbreviations

APEC: avian-pathogenic *Escherichia coli*
AMR: antimicrobial resistance
CLSI: Clinical and Laboratory Standards Institute
EMB: eosin methylene blue
ESβL: extended-spectrum beta-lactamase
ExPEC: extra-intestinal pathogenic *E. coli*
MDR: multiple drug resistance
PFGE: pulsed-field gel electrophoresis
PMQR: plasmid-mediated quinolone resistance
QRDR: quinolone resistance determining region
